# Supplementary material for: Origin, Genetic Variation and Molecular Epidemiology of SARS-CoV-2 Strains Circulating in Sardinia (Italy) during the First and Second COVID-19 Epidemic Waves
Source: Viruses. 2023 Jan 18;15(2):277. doi: 10.3390/v15020277 (PMC9961045; doi:10.3390/v15020277)
Supplement: Supplementary file 1 [file viruses-15-00277-s001.zip › Supplementary Table S2.pdf]

Supplementary Table S2: Sars-Cov-2 samples Coverage

ONT: Oxford Nanopore Technologies

Sample ID	NGS Method	Mean Coverage	Sd Coverage	Median Coverage
ITA/SS-COV-01	Illumina	1881.61	2327.93	476.00
ITA/SS-COV-02	Illumina	2191.72	2474.28	910.00
ITA/SS-COV-03	Illumina	1940.37	2359.53	624.00
ITA/SS-COV-04	Illumina	2226.57	2567.46	1170.00
ITA/SS-COV-05	Illumina	2201.52	2559.64	943.50
ITA/SS-COV-06	Illumina	2059.29	2455.94	757.00
ITA/SS-COV-07	Illumina	2159.27	2531.01	873.00
ITA/SS-COV-08	Illumina	2334.80	2601.78	1438.50
ITA/SS-COV-09	Illumina	4990.61	3097.16	6432.00
ITA/SS-COV-11	Illumina	4454.18	3043.49	4764.00
ITA/SS-COV-12	Illumina	4154.37	3039.22	3969.00
ITA/SS-COV-13	Illumina	4433.35	2935.36	4765.00
ITA/SS-COV-14	Illumina	5606.18	2959.69	7365.00
ITA/SS-COV-15	Illumina	6051.63	2483.15	7369.00
ITA/SS-COV-16	Illumina	5686.81	2711.39	7079.00
ITA/SS-COV-17	Illumina	6241.75	2523.14	7672.00
ITA/SS-COV-18	Illumina	6673.78	1959.40	7697.00
ITA/SS-COV-19	Illumina	6702.33	2143.07	7783.00
ITA/SS-COV-20	Illumina	5792.72	2804.69	7408.00
ITA/SS-COV-21	Illumina	5315.05	2910.66	6782.00
ITA/SS-COV-22	Illumina	6538.76	2184.01	7712.00
ITA/SS-COV-23	Illumina	6054.17	2617.45	7552.00
ITA/SS-COV-25	Illumina	6700.47	2106.31	7779.00
ITA/SS-COV-26	Illumina	6598.13	2263.57	7780.00
ITA/SS-COV-27	Illumina	6472.27	2271.49	7704.00
ITA/SS-COV-28	Illumina	6126.48	2363.49	7367.00
ITA/SS-COV-29	Illumina	6412.49	2300.02	7716.00
ITA/SS-COV-30	Illumina	6978.98	1829.92	7846.00
ITA/SS-COV-31	Illumina	6679.85	2105.32	7754.00
ITA/SS-COV-32	Illumina	6578.20	2140.37	7717.00
ITA/SS-COV-33	Illumina	7132.87	1655.09	7872.00
ITA/SS-COV-34	Illumina	6885.73	1966.40	7846.00
ITA/SS-COV-35	Illumina	6289.57	2334.34	7572.00
ITA/SS-COV-36	Illumina	6866.42	1822.35	7794.00
ITA/SS-COV-37	Illumina	6898.27	1789.19	7781.00
ITA/SS-COV-38	Illumina	6840.85	1956.97	7818.00
ITA/SS-COV-39	Illumina	5415.82	2850.28	6828.00
ITA/SS-COV-40	Illumina	6497.60	2274.42	7738.00
ITA/SS-COV-41	Illumina	6828.04	1926.61	7819.00
ITA/SS-COV-42	Illumina	6362.39	2302.20	7608.00
ITA/SS-COV-43	Illumina	6592.23	2121.69	7702.00
ITA/SS-COV-44	Illumina	6812.15	1929.26	7798.00
ITA/SS-COV-45	Illumina	5955.82	2624.20	7435.00
ITA/SS-COV-46	Illumina	6107.79	2536.00	7559.00
ITA/SS-COV-47	Illumina	374.08	139.05	378.00
ITA/SS-COV-48	ONT	399.89	135.69	388.00
ITA/SS-COV-49	ONT	284.08	135.35	284.00
ITA/SS-COV-50	ONT	353.82	140.11	363.00
ITA/SS-COV-51	ONT	379.21	138.50	383.00

ITA/SS-COV-52	ONT	232.18	109.29	221.00
ITA/SS-COV-53	ONT	221.09	113.25	214.00
ITA/SS-COV-54	ONT	256.97	130.92	253.00
ITA/SS-COV-55	ONT	56.73	26.65	53.00
ITA/SS-COV-56	ONT	67.22	33.72	64.00
ITA/SS-COV-57	ONT	45.66	22.06	45.00
