# Supplementary material for: Origin, Genetic Variation and Molecular Epidemiology of SARS-CoV-2 Strains Circulating in Sardinia (Italy) during the First and Second COVID-19 Epidemic Waves
Source: Viruses. 2023 Jan 18;15(2):277. doi: 10.3390/v15020277 (PMC9961045; doi:10.3390/v15020277)
Supplement: Supplementary file 1 [file viruses-15-00277-s001.zip › Supplementary Table S3.pdf]

Supplementary Table S3. Amino acid mutations detected in the SARS-CoV-2 proteins. The number of samples presenting a specific mutation is reported in brackets. The percentages of the mutations for each protein is reported in the box below.

<table>
<tr><th colspan="14">ORF1ab</th><th></th><th>ns3</th><th></th><th>ns8</th><th></th></tr>
<tr><th colspan="14"></th><th>S</th><th></th><th>ns7a</th><th></th><th>N</th></tr>
<tr><th>nsp1</th><th>nsp2</th><th>nsp3</th><th>nsp4</th><th>nsp5</th><th>nsp6</th><th>nsp7</th><th>nsp9</th><th>nsp10</th><th>nsp12</th><th>nsp13</th><th>nsp14</th><th>nsp15</th><th>nsp16</th><th></th><th></th><th></th><th></th><th></th></tr>
<tr>
<td>F143I(1)
K141Q(1)
L140del(1)
S142V(1)</td>
<td>H194Y(1)
I273T(2)
L289F(1)
P191L(2)
Q496H(27)
T170I(1)</td>
<td>A358T(1)
A890D(1)
D9G(1)
E159G(3)
I1412T(1)
I1683T(2)
I385T(1)
I868L(1)
P1442L(1)
S1534G(1)
T183I(1)
V1229F(1)</td>
<td>A380V(1)
L438I(1)
M324I(2)
V103A(6)</td>
<td>M162I(1)
T304I(6)</td>
<td>C221F(1)
F108del(1)
G107del(1)
L260F(1)
L37F(1)
S106del(1)</td>
<td>D77Y(1)</td>
<td>M101I(2)</td>
<td>T101I(1)</td>
<td>A185S(2)
A97V(1)
L186F(1)
P323L(55)
T26I(1)
V720I(2)
V776L(2)</td>
<td>A267S(4)
A389T(1)
A505V(1)
A598S(2)
E261D(2)
H290Y(2)
K218R(2)
S74P(1)
V60L(2)</td>
<td>A435V(6)
A96V(1)
P297L(1)
P46S(2)</td>
<td>L162F(1)
S287L(1)</td>
<td>S33I(1)
T93M(1)</td>
<td>A222V(44)
A262S(8)
A570D(1)
A67V(1)
A771S(1)
D118H(1)
D614G(55)
E1195A(1)
H69del(3)
L1203F(1)
L822F(1)
N439K(2)
N501Y(1)
P272L(8)
P631S(1)
P681H(1)
Q677H(1)
Q836L(1)
S477N(2)
S982A(1)
T716I(1)
T791I(1)
V70del(3)
Y144F(28)
YI44del(1)</td>
<td>A54S(1)
G100C(1)
N256D(1)
Q57H(2)
S26L(2)
V202L(1)
V255del(1)
V97L(1)</td>
<td>E95stop(1)
G38stop(1)</td>
<td>P70S(4)
Q27stop(1)
R52I(1)
T87I(2)
Y73C(1)</td>
<td>A182S(1)
A220V(45)
A35E(6)
A376T(2)
D3L(1)
G204L(1)
G204R(6)
H145Y(3)
M234I(2)
P46S(2)
Q389H(1)
R203E(6)
R203K(6)
S235F(1)
T141I(2)</td>
</tr>
<tr><td>3.5%</td><td>5.2%</td><td>10.3%</td><td>3.5%</td><td>1.7%</td><td>5.2%</td><td>0.9%</td><td>0.9%</td><td>0.9%</td><td>6%</td><td>7.8%</td><td>3.4%</td><td>1.7%</td><td>1.7%</td><td>21.6%</td><td>6.9%</td><td>1.7%</td><td>4.3%</td><td>12.9%</td></tr>
</table>
